# Supplementary material for: The Passage of Chaperonins to Extracellular Locations in Legionella pneumophila Requires a Functional Dot/Icm System
Source: Biomolecules. 2025 Jan 9;15(1):91. doi: 10.3390/biom15010091 (PMC11763710; doi:10.3390/biom15010091)
Supplement: Supplementary file 1 [file biomolecules-15-00091-s001.zip › Supplemental Table S1.pdf]

Table S1. Primers used. Boldface letters in the sequence box indicate where the corresponding restriction site named in column three is located. Underlined text in the sequence box of primer P26 shows the reverse six histidine codons added to the *htpB* 3′ end.

Primer code	Sequence (5′ to 3′)	Restriction site	Use
P1	CAATTA**GCGGCCGC**AGGAACCAACCCATATC	*Not*I	F-*dotA* upstream
P2	GGGCGC**GGATCC**CATTTCAGGAGTAGGATTAC	*Bam*HI	R-*dotA* upstream
P3	GGACGC**GGATCC**CAATCGGATCAAGGAATAG	*Bam*HI	F-*dotA* downstream
P4	CCGGGC**CTCGAG**CTCTGAAAGGAGTCATTAAA	*Xho*I	R-*dotA* downstream
P5	CCTGACCCTAAACGTGCTTAT	NONE	F-*dotA* internal
P6	GAACTGACCACGGCCATATT	NONE	R-*dotA* internal
P7	GGCCCG**GTCGAC**ATAATGGCTAAAGAATTACG	*Sal*I	F-*htpB* for GSK fusion
P8	CCTATT**GCATGC**TTACATCATTCCGCCCATGC	*Sph*I	R-*htpB* for GSK fusion
P9	GCGAAT**GTCGAC**TCTCATTCCAAAAGAGAAC	*Sal*I	F-*legC6* for GSK fusion
P10	GTTATG**GTCGAC**TTATTTTGGTAATGAACCGAG	*Sph*I	R-*legC6*
P11	GTAGCC**GTCGAC**ACAAATAATCGAGTAAGAG	*Sal*I	F-*mdh* for GSK fusion
P12	TGAGAT**GTCGAC**TTAATCCAATAAGCCTAAAGAC	*Sph*I	R-*mdh*
P13	GCCGGG**GCGGCCGC**GAGCTAATTTAGCT	*Not*I	F-*icdH*
P14	GCGGGC**GGATCC**CATATGCTTAATCATGGCATCTG	*Bam*HI	R-*icdH* with no stop codon
P15	GCGGGC**GGATCC**CGTATGGCTCTGCAAA	*Bam*HI	F-*htpB* last 150 base pairs
P16	GCGGGC**GGATCC**ATGCGTCAGATTGTTA	*Bam*HI	F-*htpB* last 300 base pairs
P17	CCGGGG**CTCGAG**AGTATCAGCAAGAGC	*Xho*I	R-*htpB* for 150/300 base pairs
P20	GGCCGCGGATCCTTACTGTTACTAATAAACATTATTGTAGAC TCTGT	NONE	Sequencing primer for all GSK constructs
P25	GGCCGC**GCATGC**CATTCGATATCTTTTACTGTTACTAATAAAC ATTATTGTAGAC	*Sph*I	F-*htpB* for 6His tag construct
P26	TTATAT**TCTAGA**TTA<u>ATGATGATGATGATGATG</u>TCCTGCCATC ATTCCGCCCATGCCAC	*Xba*I	R-*htpB* contains the codons for the 6His tag
P27	GGGGGAAAAGATTTTTGTGAGGACG	NONE	F-*enhA* positive PCR control
P28	GGGAACTTCATAGGCAGCATGTATT	NONE	R-*enhA* positive PCR control

Abbreviations: F = Forward primer, R = Reverse primer
