# Supplementary material for: The Passage of Chaperonins to Extracellular Locations in Legionella pneumophila Requires a Functional Dot/Icm System
Source: Biomolecules. 2025 Jan 9;15(1):91. doi: 10.3390/biom15010091 (PMC11763710; doi:10.3390/biom15010091)
Supplement: Supplementary file 1 [file biomolecules-15-00091-s001.zip › Supplemental Table S2.pdf]

SupplementalTable S2. Densitometry data of immunostained ICDH, HtpB and DsbA2 bands, in lysates or concentrated shockates, used to estimate the percent ratio of true periplasmic HtpB or DsbA2 in different fractionated *L. pneumophila* Lp02 derivatives.

	Pt conc mg/mL	Vol. mL	Total mg	ICDH IOD	ICDH[1] OD/μg	ICDH Tot. OD	% cont.	HtpB IOD	HtpB[1] OD/μg	HtpB Tot. OD	% Per. HtpB[2]	DsbA2 IOD	DsbA2[1] OD/μg	DsbA2 Tot. OD	% Per. DsbA2[2]
Lp02 L1	6.25	5	31.25	165	3.3	103,125	0.86	80.3	1.6	50,000	0	ND	ND	ND	ND
Lp02 S1	0.25	0.25	0.063	161	14.3	891		54.4	4.8	302		ND	ND	ND	
dotB^{-} L1	11.75	5	58.75	269	5.7	334,875	0.30	78.3	1.7	99,875	0.07	ND	ND	ND	ND
dotB^{-} S1	1.125	0.25	0.281	179	3.5	994		65.5	1.3	365		ND	ND	ND	
Lp02 L2	5.73	5	28.64	83.3	2.8	80,192	0.06	160.1	5.3	151,818	0.11	91.2	3.0	85,920	0.46
Lp02 S2	391	0.17	0.067	20.0	0.67	44.9		120.6	4.0	266		200.3	6.7	449	
dotB^{-} L2	12.11	5	60.55	44.8	1.5	90,825	0.14	152.3	5.1	308,779	0.24	74.1	2.5	151,375	0.59
dotB^{-} S2	2.083	0.14	0.292	13.0	0.43	125.6		120.5	4.0	1,166		113.4	3.8	1110	
dotB^{-} L3	19.30	5	96.5	80.0	4.0	386,000	0.17	181.6	9.1	878,150	0.04	115.6	5.8	559,700	0.36
dotB^{-} S3	7.28	0.08	0.582	22.8	1.1	640		64.8	3.2	1,862		102.8	5.1	2,968	
+ V L	10.76	5.5	59.18	63.6	3.2	189,376	0.23	104.6	5.2	307,736	0.26	55.1	2.8	165,704	0.46
+ V S	12.66	0.05	0.633	14.0	0.7	443		48.9	2.4	1,519		35.0	1.8	1,139	
+ C L	4.43	15	66.45	43.8	2.2	146,190	0.56	127.5	6.4	425,280	0	42.0	2.1	139,545	0.69
+ C S	5.38	0.17	0.915	17.9	0.9	823		49.5	2.5	2,288		37.6	1.9	1,739	

[1] The OD/μg values for the ICDH, HtpB, and DsbA2 immunoblots, were calculated by dividing the IOD value of each band, by the amount of protein loaded per lane (in μg) of the corresponding SDS-PAGE gel. For the 1st experiment, the μg/lane were: Lp02 L1=50 μg, Lp02 S1=11.3 μg, *dotB*$^{-}$ L1=47 μg, *dotB*$^{-}$ S1=50.6 μg. For the 2nd experiment, all lanes were loaded with 30 μg total protein, and for the 3rd experiment all lanes were loaded with 20 μg total protein.

[2] The % values shown for periplasmic HtpB or DsbA2, have been already corrected by subtracting the % contamination.

Abbreviations: L = lysate, S = shockate, +V = *dotB*$^{-}$ + V (empty vector), +C = *dotB*$^{-}$ + C (*trans*-complementing plasmid), Pt = protein, conc = concentration, Vol. = volume, cont. = contamination, Per. = periplasmic, ND = Not determined
