# Supplementary material for: The Passage of Chaperonins to Extracellular Locations in Legionella pneumophila Requires a Functional Dot/Icm System
Source: Biomolecules. 2025 Jan 9;15(1):91. doi: 10.3390/biom15010091 (PMC11763710; doi:10.3390/biom15010091)
Supplement: Supplementary file 1 [file biomolecules-15-00091-s001.zip › Supplementary Figures.pdf]

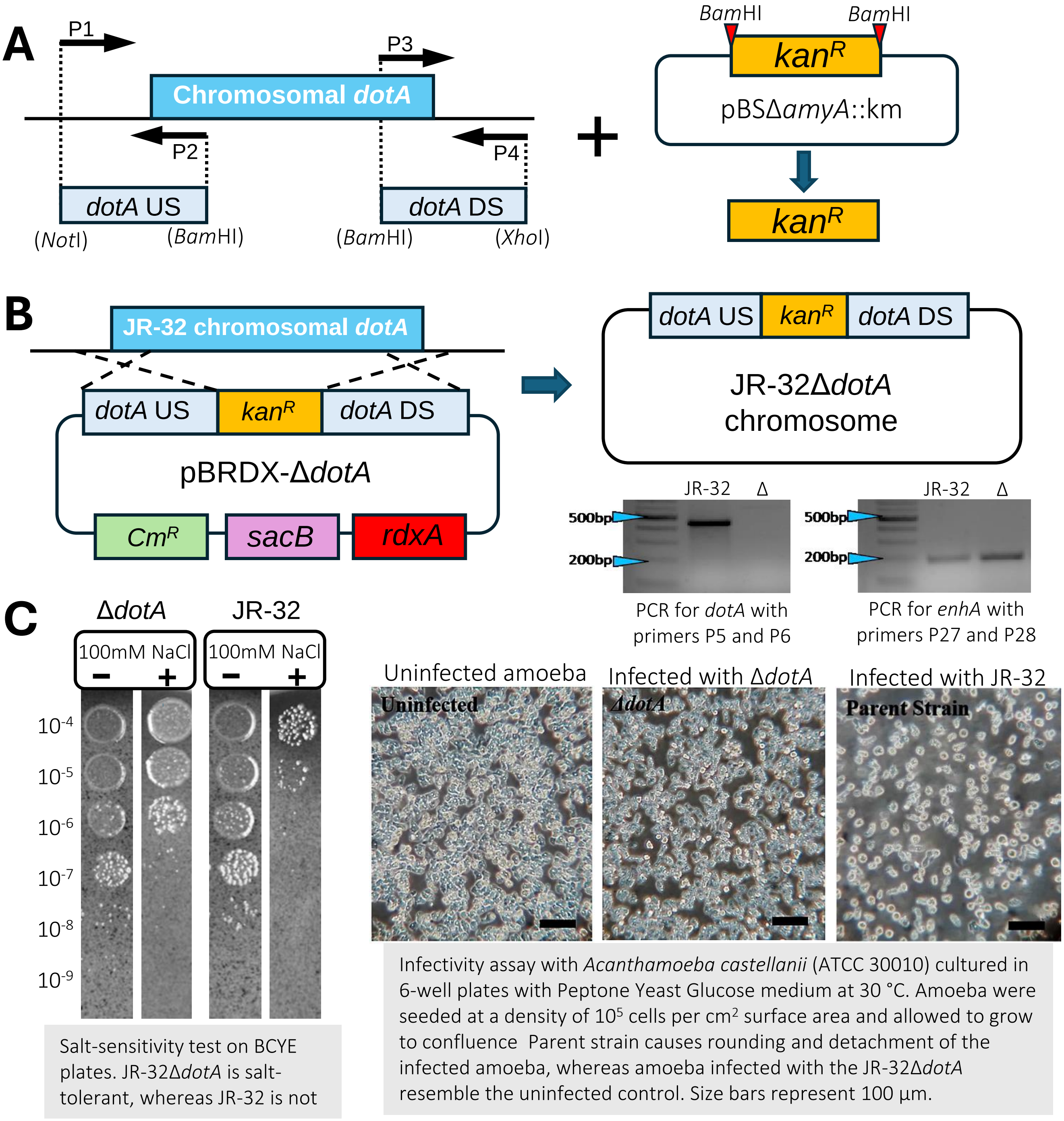

Figure S1. Creation of the JR-32Δ*dotA* mutant. (**A**) The upstream (US) and downstream (DS) regions of *dotA* were amplified by PCR using primers P1, P2, P3, and P4. The amplification products were cut with the restriction enzymes indicated, and sequentially ligated to the Km3 kanamycin-resistance cassette [42] dropped from plasmid pBSΔ*amyA*::km (from Dr. H. Abdelhady, Dalhousie University). (**B**) The construct from panel A was cloned into suicide plasmid pBRDX for the allelic replacement of *dotA*. A Kan^R, Cm^S, sucrose-resistant and metronidazole-resistant clone was tested by PCR to ensure the lack of an intact *dotA* internal sequence. The PCR of *enhA* demonstrates that the lack of amplification of *dotA* was not due to lack of DNA template. Δ = JR-32Δ*dotA*. (**C**) Phenotypic characterization of JR-32Δ*dotA*. A serially diluted suspension of bacteria was spotted on BCYE with (+) or without (-) 100 mM NaCl. Plates were incubated at 37 °C for 3 days before they were photographed. Amoeba in 6-well plates were infected at a bacteria:cell ratio of 100:1 and incubated at 30 °C for 16 hours before micrographs were taken in an Olympus BX6 microscope.

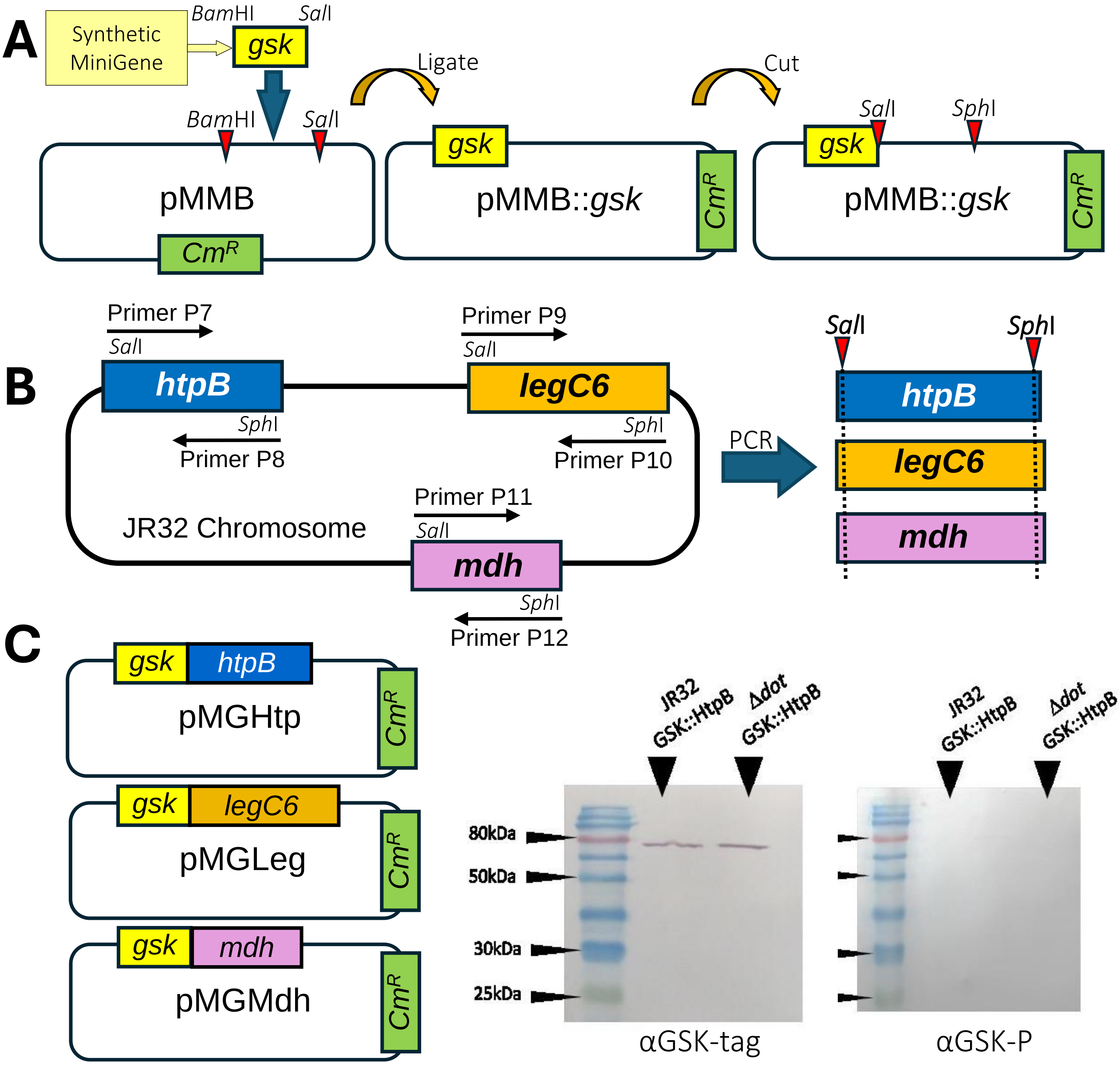

Figure S2. Construction of plasmids for the expression of GSK-tagged proteins. (**A**) A synthetic G-block (Integrated DNA Technologies Inc.) encoding a 13-amino acid peptide derived from the eukaryotic protein Glycogen Synthase Kinase (GSK) and the indicated restriction sites was cloned in plasmid pMMB to construct pMMB::*gsk*, which was then cut with *Sal*I and *Sph*I (red triangles). (**B**) The sequences of *htpB* (*lpg0688*), *legC6* (*lpg1588*), and *mdh* (*lpg2352*) were amplified by PCR with the indicated primers encoding the restriction sites shown by the arrows, and subsequently cut with the *Sal*I and *Sph*I restriction enzymes. (**C**) The cut amplification products from panel B were ligated into the cut pMMB::*gsk* from panel A to create the three working plasmids pMGHtp, pMGLeg and pMGMdh. The correctness of the constructs was confirmed by unidirectional DNA sequencing (contracted from Genome Quebec - Montreal PQ, Canada) with primer P20. The expression of the recombinant tagged proteins was confirmed by immunoblotting, as shown in the case of GSK-HtpB. The membranes shown were immunostained with the antibodies indicated at the bottom of the panel.

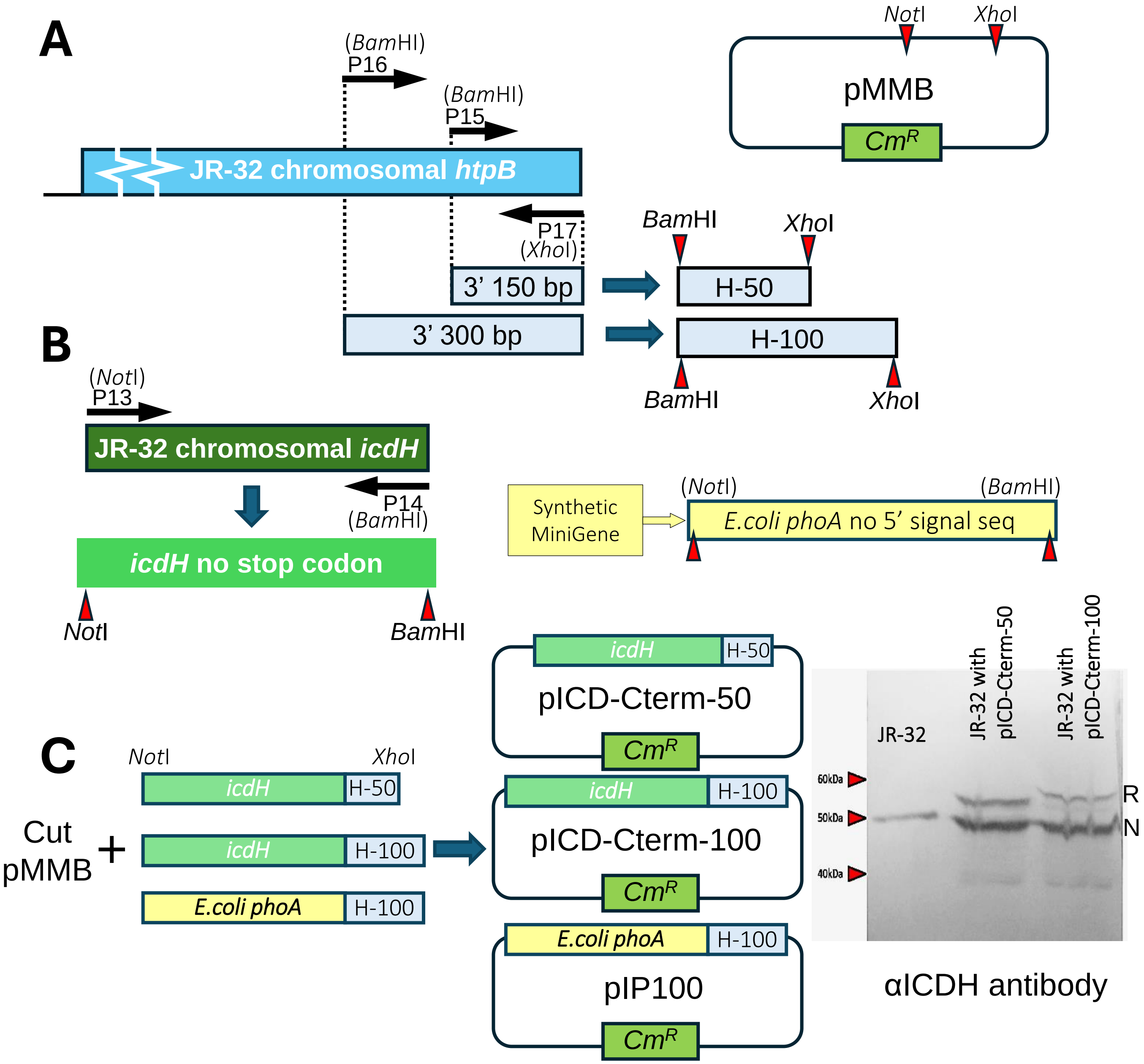

Figure S3. Construction of plasmids encoding fusions of ICDH or PhoA with the C-term of HtpB. (**A**) The 3' end of *htpB* (last 150- or 300-base pairs) was amplified by PCR with the indicated primers encoding the restriction sites shown. The amplification products were cut with *Bam*HI and *Xho*I, as was plasmid pMMB with *Not*I and *Xho*I. (**B**) The *icdH* gene was amplified by PCR with primers P13 and P14, and the amplification product cut with *Not*I and *Bam*HI. These same restriction sites were used to cut a synthetic G-block (Integrated DNA Technologies Inc.) encoding the *E. coli phoA* gene without the sequence corresponding to the PhoA N-term signal sequence required for secretion, and also, no stop codon. (**C**) The cut fragments from panel A and panel B were separately ligated to create *icdH*::H-50, *icdH*::H100, and *phoA*::H100, which were then cloned into the cut plasmid pMMB from panel A to create the working plasmids pICD-Cterm-50, pICD-Cterm-100, and pIP100. The expression of the two recombinant ICDH forms was confirmed by immunoblotting with antibody αICDH. It should be noted that JR-32 carrying the pICD-Cterm plasmids produces both the native (N) and the recombinant (R) forms of ICDH.

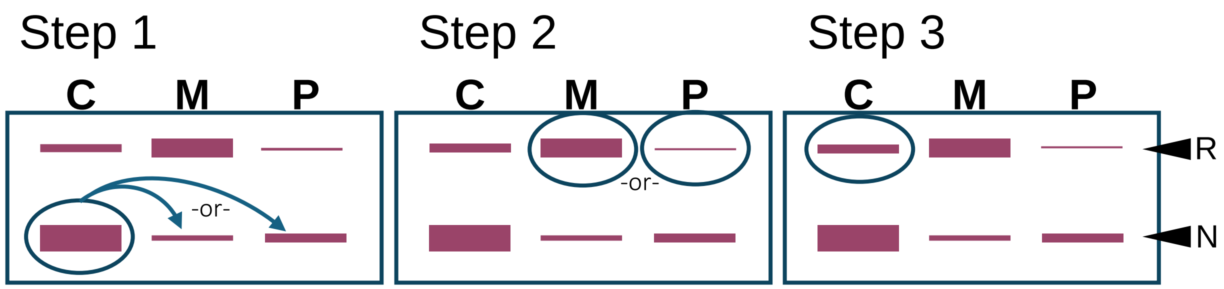

Step 1

For membrane:
Divide density of native ICDH in cytoplasm / density of native ICDH in the membrane fraction
For periplasm:
Divide density of native ICDH in cytoplasm / density of native ICDH in the periplasm fraction

Step 2

For membrane:
Take the density of recombinant ICDH in the membrane fraction
For periplasm:
Take the density of recombinant ICDH in the periplasm fraction

Step 3

For both membrane and periplasm:
Take the density of recombinant ICDH in the cytoplasm

$$TSR_{membr} = 1 - \frac{ACS}{HPS_{membr}}$$

$$TSR_{peripl} = 1 - \frac{ACS}{HPS_{peripl}}$$

ACS = Density of recombinant ICDH in the cytoplasm fraction (from step 3)

HPS = Ratio from step 1 for membrane or periplasm × Density of recombinant ICDH in membrane or periplasm (from step 2)

Figure S4. Diagrammatic explanation of the method for calculating the True Secretion Ratio (TSR). The TSR was calculated for either the membrane or the periplasm fraction, using the formulas given, which take into account the relative abundance of recombinant ICDH forms (either carrying the last 50- or 100-amino acids of the HtpB C-term) in relation to the native ICDH present in the corresponding cellular fraction. Because native and recombinant ICDH forms are detected with the same antibody in the same sample lane, native ICDH serves as an internal localization/contamination control. Abbreviations: C = cytoplasm fraction, M = membrane fraction, P = periplasm fraction, R = recombinant ICDH form, N = native ICDH, membr = membrane, peripl = periplasm, TSR = true secretion ratio, ACS = actual cytoplasmic recombinant protein seen, HPS = hypothetical recombinant protein secreted.
